# DL-3-n-butylphthalide improves cerebral hypoperfusion in patients with large cerebral atherosclerotic stenosis: a single-center, randomized, double-blind, placebo-controlled study

**DOI:** 10.1186/s12883-020-01801-5

**Published:** 2020-05-26

**Authors:** Dawei Chen, Yanwei Yin, Jin Shi, Fen Yang, Kehua Wang, Faguo Zhao, Wenping Li, Bin Li

**Affiliations:** 1 Department of Neurology, Air Force Medical Center, PLA (People’s Liberation Army), 30# Fucheng Road, Haidian District, Beijing, 100142 China; 2 PET Center, Air Force Medical Center, PLA (People’s Liberation Army), 30# Fucheng Road, Haidian District, Beijing, 100142 China

**Keywords:** DL-3-n-butylphthalide, Cerebral atherosclerotic stenosis, Cerebral hypoperfusion, Cerebral blood flow, Single-photon emission computed tomography

## Abstract

**Background:**

DL-3-n-butylphthalide (NBP) was demonstrated to increase the cerebral blood flow (CBF) in the animal models, but there are no clinic studies to verify this. We aimed to explore the effect of NBP on improving cerebral hypoperfusion caused by cerebral large-vessel stenosis.

**Methods:**

In this single-center, randomized, double-blind, placebo-controlled study, 120 patients with severe carotid atherosclerotic stenosis and cerebral hypoperfusion in the ipsilateral middle cerebral artery (MCA) were included and randomly assigned into NBP or placebo group as 1:1 radio. Patients in NBP or placebo group received 200 mg or 20 mg of NBP capsules three times daily for four weeks respectively. Single photon emission computed tomography (SPECT) was used to assess regional CBF (rCBF) in four regions of interest (ROIs) corresponding to MCA before and 12 weeks after the treatment. After therapy, the rCBF change for every ROI and the whole CBF change in MCA territory for every patient were classified into amelioration, stabilization and deterioration respectively.

**Results:**

48 NBP patients (6 with bilateral stenosis) and 46 placebo patients (8 with bilateral stenosis) completed the trial. Overall, both groups had 54 stenotic carotid arteries and 216 ROIs for rCBF change analysis. After therapy, the rCBF in ROIs increased in NBP group (83.5% ± 11.4% vs. 85.8% ± 12.5%, *p* = 0.000), whereas no change was found in placebo group (86.9% ± 11.6% vs. 87.8% ± 11.7%, *p* = 0.331). Besides, there was higher percentages of ROIs with rCBF amelioration and stabilization in NBP group than in placebo group (93.1% vs. 79.2%, *p* = 0.000). Furthermore, ordinal regression analysis showed that compared with placebo, NBP independently made more patients to have whole CBF amelioration in ipsilateral MCA (Wald-χ2 = 5.247, OR = 3.31, *p* = 0.022).

**Conclusions:**

NBP might improve the cerebral hypoperfusion in the patients with carotid artery atherosclerotic stenosis.

**Trial registration:**

Chinese Clinical Trial Registry, ChiCTR1900028005, registered December 8th 2019- Retrospectively registered (http://www.chictr.org.cn/index.aspx).

## Background

Hypoperfusion caused by large intracranial and extracranial atherosclerotic stenosis can impair clearance of distal emboli, which is an alternative important mechanism of ischemic stroke, in addition to thromboembolism [1, 2]. Approximal 20% ~ 30% of ischemic strokes in the United States were attributed to cerebral large-vessel stenosis [3]. The study of China National Stroke Registry showed that large arterial atherosclerotic stenosis accounted for 45% ischemic strokes in China [4]. In addition, many studies showed that chronic cerebral hypoperfusion (CCH) was associated with a higher prevalence of cognitive impairment [5]. Therefore, improving cerebral hypoperfusion may prevent ischemic stroke and cognitive impairment. Although carotid endarterectomy (CEA) and carotid artery stenting (CAS) can normalize cerebral hemodynamics by increasing the lumen diameter and providing a better cerebral irrigation, they are not suitable to all the patients with cerebral large-vessel stenosis [6]. The augmentation of collateral vessels may also enhance the cerebral blood flow in these patients. Some pharmacologic approaches such as volume expansion, hemodilution, vasodilatation, and induced hypertension, have been suggested to increase collateral perfusion, but unfortunately, the trials of these drugs failed to demonstrate an overall clinical benefit among the participants [7]. Consequently, 2019 American Heart Association’s guidelines for ischemic stroke advised against the common use of these medicines to enhance collateral circulation [8].

Dl-3-butylphthalide (NBP) is a synthetic chiral compound based on l-3-butylphthalide, which is originally isolated from seeds of *Apium graveolens* [9]. The systematic review showed that the combination of NBP and standard anti-ischemic stroke agents was superior to standard drugs alone in the therapy of patients with acute ischemic stroke, based on both the Barthel index and National Institutes of Health Stroke Scale, as a neuroprotective drug [10]. The numerous animal studies also showed that NBP decreased the ischemic brain area and improved the neurological deficits. NBP could improve the cerebral microcirculation and cerebral blood flow (CBF) by vasodilatation and angiogenesis in these animal studies [11–14]. However, there are still no clinic studies to verify whether NBP can improve the decreased CBF in the human being.

This clinical trial was carried out to explore the effect of NBP capsules on improving cerebral hypoperfusion in the ipsilateral middle cerebral artery (MCA) territory caused by the severe carotid atherosclerotic stenosis. This study may provide an evidence-based clinical strategy to prevent ischemic stroke and vascular cognitive impairment from cerebral large-vessel stenosis and hypoperfusion.

## Methods

### Study design

This was a single-center, randomized, double-blind, placebo-controlled trial that enrolled 120 patients from the Air Force Medical Center PLA (People’s Liberation Army) in Beijing city between January 2017 and March 2019. The trial was approved by the Institutional Review Boards in the Air Force Medical Center PLA. Participants provided written informed consent to participate in the study. This study was registered in the Chinese Clinical Trial Registry (No. ChiCTR1900028005).

### Patients

The patients, who had both severe atherosclerotic stenosis in the carotid artery and cerebral hypoperfusion in the ipsilateral MCA territory, were invited to participate in this study by the trial investigators. Eligibility criteria included the following: (1) 30 ~ 80 years of age; (2) ≥70% stenosis or occlusion in the internal carotid artery (ICA) and/or the first sphenoidal segment (M1) of MCA; (3) cerebral hypoperfusion in the ipsilateral MCA territory; (4) no transient ischemic attacks (TIA) or ischemic strokes within 2 weeks; (5) no ≥50% stenosis or occlusion in the subclavian artery, vertebral artery, basilar artery and posterior cerebral artery; (6) none or ≤ 15 mm infarction in the territory of MCA and cerebellum on CT or MRI. We excluded the following subjects:(1) cerebral stenosis caused by any disease other than atherosclerosis, such as autoimmune disorder and vasculitis; (2) preferring to carry out CAS or CEA; (3) contraindications for assessing cerebral stenosis and perfusion; (4) other cerebral diseases (such as infection, degeneration, demyelination, tumor and trauma) which influence cerebral perfusion; (5) pregnant or lactating women; (6) severe cardiac, pulmonary, hepatic or renal diseases and life expectancy ≤6 months; (7) psychotic; (8) allergy to NBP.

### Randomization and drug treatment

Patients were randomly assigned in a 1:1 ratio to NBP and placebo groups. Both groups received three times daily oral NBP 200 mg or 20 mg (ineffective dose) for 4 weeks respectively. At same time all the patients were required to receive optimized medical therapy (OMT) against cerebral artery atherosclerotic stenosis, including antiplatelet aggregation, lipid-lowering, antihypertension and hypoglycemia in their whole life, according to 2018 Chinese stroke guidelines [15]. However, any vascular dilation drug was contraindicated for the patients.

An independent statistician from Chinese Beijing Medical University created the randomization list by the compute. Every kit of drug was labeled with sequential numbers corresponding to the randomization list. The successive patients were distributed with the kits from lowest number to highest number by the drug administrator. All kits of drugs had identical appearance and similar smell. The patients, investigators and drug administrator were blinded to the drug allocation.

### Clinical assessment and follow-up

Initial assessments of the patients before the therapy included demographic characters, the atherosclerotic risk factors (hypertension, diabetes mellitus, hyperlipidemia, coronary heart disease and smoking), cerebral stenotic location and degree, integrity of the circle of Willis (CoW), and CBF. The cerebral artery stenosis and integrity of CoW were assessed by magnetic resonance angiography (MRA), computed tomography angiography (CTA), or digital subtraction angiography (DSA). The extracranial artery stenotic degree was calculated according to North American Symptomatic Carotid Endarterectomy Trial Collaborators (NASCET) and the intracranial artery stenotic degree was calculated according to Warfarin-Aspirin Symptomatic Intracranial Disease (WASID). CBF was assessed by single photon emission computed tomography (SPECT). The medication compliance of NBP and its adverse reactions were followed up by telephone after 4 weeks. CBF was assessed again after 12 weeks.

### SPECT procedures and region of interest (ROI) selection

SPECT brain scans were carried out in a dark and quiet environment according to our previous study [6]. 925 MBq of ethylcysteinate dimer (^99m^TC-ECD, China institute of atomic energy, China) was injected into the antecubital vein. The images were taken by the SPECT scanner (Infinia Hawkeye; General Electric Company, USA) after one hour. The images were taken per 6° during rotation of 360° and at a rate of 35 s per frame. The data were acquired on a 128 × 128 matrix.

ROI selection and data analysis were also according to previous studies [6, 16]. NeuroMatch Software package (GE Medical Systems; Segami Corporation, Columbia, MD, USA) was used for the ROI selection. Four slices of cerebral hemisphere were selected on orbitomeatal line (OML) + 55 mm, + 58 mm, + 61 mm, and + 64 mm.Six pairs of sphenoid mirrored ROIs over cortical gray matter were drawn at each slice of cerebral hemisphere. Four middle ROIs corresponding to the MCA territory were selected, and the mean value of four slices in each ROI was used for data analysis. However, mirrored ROIs were also drawn in one slice of cerebellar hemisphere (OML + 15 mm) for normalization of cerebral hemodynamic parameters.

### Definition of CBF and cerebral hypoperfusion

The ratio of radioactive counting obtained from the ROI in the cerebral hemisphere to that from the ROI in the ipsilateral cerebellar hemisphere was calculated automatically by computer for the cerebral hemodynamic normalization, which was defined as CBF. The CBF in each ROI was called as regional CBF (rCBF). The cerebral hypoperfusion was defined as rCBF_stenosis_ ≤ 90% and/or rCBF_mirror_-rCBF_stenosis_ ≥ 90% in any ROI corresponding to the ipsilateral MCA territory [16].

### Outcome measures

The primary efficacy outcome was the proportion of ROIs with different rCBF change at 12 weeks after treatment. As for a ROI in the ipsilateral MCA of carotid artery stenosis, its rCBF change was classified according to the difference between pre-treatment and post-treatment rCBF. The rCBF amelioration was defined as rCBF_after_ - rCBF_before_ ≥ 10%, and the rCBF deterioration was defined as rCBF_after_ - rCBF_before_ ≤ − 10%, and the rCBF stabilization was defined as − 10%<rCBF_after_ - rCBF_before_<10% [16]. The secondary outcome was the proportion of patients with different whole CBF change at 12 weeks after treatment. As for a patient with carotid artery stenosis, the whole CBF change in the ipsilateral MCA territory was classified according to the number of ROIs with rCBF deterioration or amelioration after treatment (4 ROIs in the unilateral stenosis and 8 ROIs in the bilateral stenosis). First, the whole CBF deterioration was defined when a patient had ≥1, ≥2, ≥3 or ≥ 4 ROIs with rCBF deterioration in the ipsilateral MCA territory. Correspondingly, the whole CBF amelioration was defined when a patient had ≥1, ≥2, ≥3 or ≥ 4 ROIs with rCBF amelioration in the ipsilateral MCA territory. At last, the whole CBF stabilization was defined in the other patients. The secondary outcome also included rCBF change extent in every ROI at 12 weeks after treatment.

### Statistical analysis

The sample size was calculated according to the primary outcome. The percentage of ROIs with rCBF improvement after OMT treatment was about 30% in our previous study [17]. A total of 196 ROIs for each group were estimated to detect a 15% difference in the proportion of ROIs with rCBF amelioration between NBP and placebo groups, achieving a power of 90% and two-sided significance levels of 0.05. Correspondingly, 100 patients (50 patients in each group) were needed since one patient with unilateral carotid artery stenosis had 4 ROIs in the MCA territory. Given an expected dropout rate of 20%, we stipulated that the total number of patients to be randomized was 120.

Continuous variables with normal distribution were presented as mean and standard deviation (SD), and continuous variables with non-normal distribution as median and interquartile range. Categorical variables were reported as the percentage of patients in the subgroup. In univariate analysis, the Student t test or the Mann-Whitney U test was used for two class comparisons of continuous variable, and one-way ANOVA or Kruskal Wallis H test for multiple-class comparisons of continuous variables. χ^2^ test was used for the comparisons of categorical variables. Multivariable ordinal regression models were performed with the whole CBF change after treatment as dependent variable and NBP as independent variable. The statistical significance was defined as *p* ≤ 0.05. All the resultant data were analyzed by the software of SPSS 16.0 (IBM, Amon, New York, USA).

## Results

**Fig. 1:**
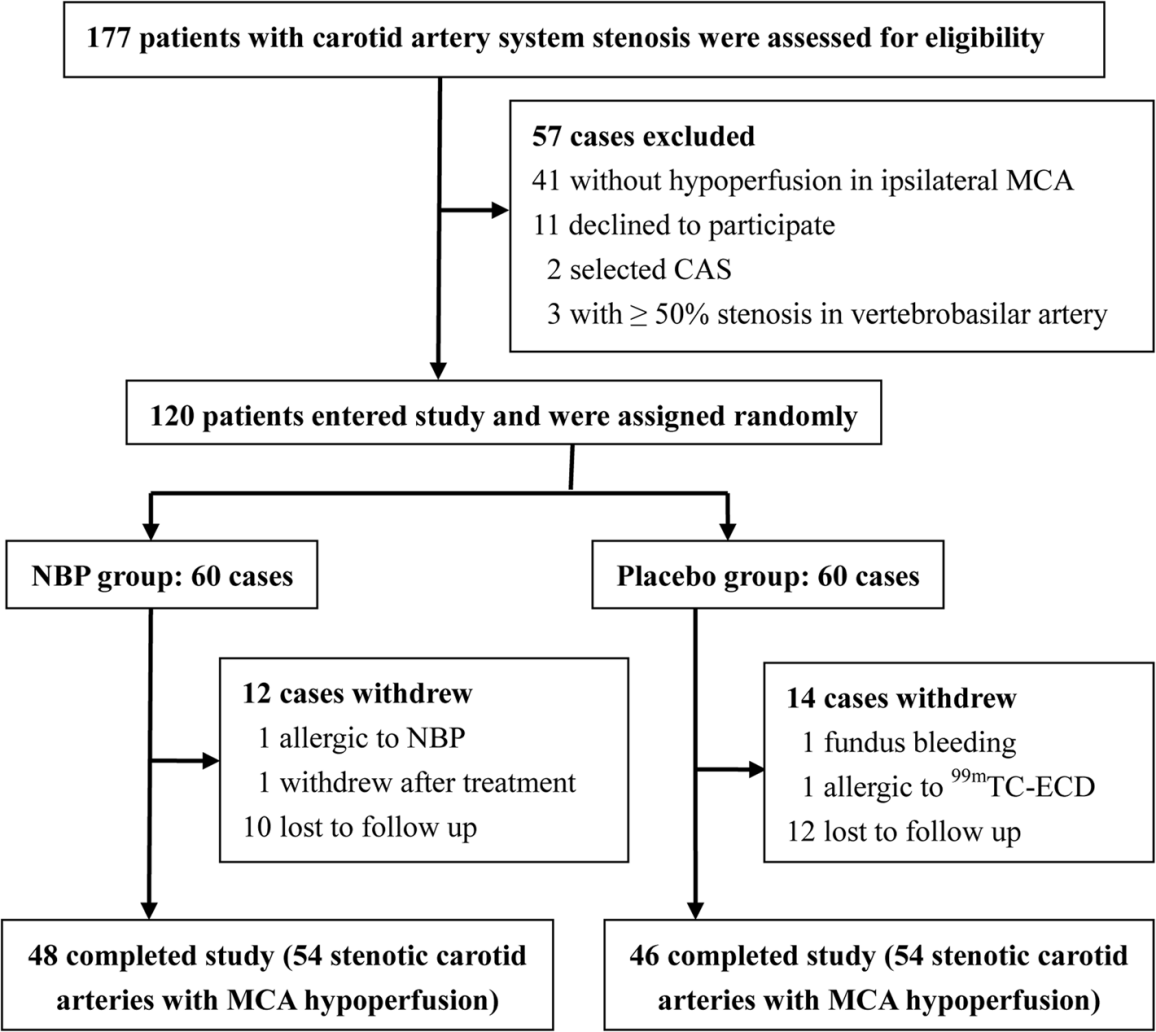
Trial profile about patient’s recruitment, participation and attrition. CBF, cerebral blood flow; MCA, middle cerebral artery; CAS, carotid artery stenting; NBP, Dl-3-butylphthalide

A total of 120 patients with cerebral hypoperfusion in the ipsilateral MCA of carotid artery stenosis, participated in this study and underwent randomization. Among them, 60 were assigned to NBP group and 60 were assigned to placebo group. As shown in Fig. 1, 48 patients among NBP group and 46 patients among placebo group completed treatment and follow-up. The trial was stopped in the following conditions: (1) the patients could not continue NBP therapy because of severer drug adverse reactions (allergic to NBP and fundus bleeding); (2) the patients could not complete the second CBF assessment because of the event that restrained the SPECT test (allergic to ^99m^TC-ECD); (3) the patients were unwilling to be followed up and have a second SPECT test. Finally, 94 patients were included in the efficacy analysis and 26 patients (21.7%) were lost to follow up in this study. Baseline demographics and clinical characteristics were well-balanced between NBP group and placebo group, which were listed in Table 1. Although all the patients were required to receive OMT from the trial beginning, eight patients (8.5%) had discontinued anti-platelet aggregation and/or lipid-lowering drugs at the 12-week follow-up evaluation. However, there was no difference in the discontinuation of OMT between NBP group and placebo group (Table 1).

**Table 1:** Baseline characteristics of 94 patients for the efficacy analyses

Characteristics	NBP group(*n* = 48)	Placebo group(*n* = 46)	*P* values
Age (years)	56.6 ± 11.5	60.6 ± 10.3	0.085
Gender (male)	39 (81.2%)	32 (69.6%)	0.188
Hypertention (yes)	38 (79.2%)	39 (84.8%)	0.479
Diabetes mellitus (yes)	19 (39.6%)	22 (47.8%)	0.420
Hyperlipemia (yes)	37 (77.1%)	38 (82.6%)	0.505
Coronary disease (yes)	8 (16.7%)	12 (26.1%)	0.265
Smoking (yes)	29 (60.4%)	24 (52.2%)	0.420
Previous TIA/ ischemic stroke			
Previous TIA	8 (16.7%)	9 (19.6%)	0.600
Previous ischemic stroke	24 (50.0%)	26 (56.5)	
None	16 (33.3%)	11 (23.9%)	
Methods for assessing stenosis			
MRA	35 (72.9%)	30 (65.2%)	0.316
CTA	1 (2.1%)	0 (0%)	
DSA	12 (25.0%)	16 (34.8%)	
Unilateral or bilateral			
Right side	20 (41.7%)	22 (47.8%)	0.526
Left side	22 (45.8%)	16 (34.8%)	
Bilateral	6 (12.5%)	8 (17.4%)	
Stenotic location			
Extracranial ICA	10 (20.8%)	8 (17.4%)	0.118
Intracranial ICA	23 (47.9%)	31 (67.4%)	
MCA(M1 segment)	15 (31.2%)	7 (15.2%)	
Carotid stenotic degree			
Severe stenosis(70–99%)	35 (72.9%)	40 (87.0%)	0.090
Occlusion	13 (27.1%)	6 (13.0%)	
Integrity of CoW			
Integrity	11 (22.9%)	17 (37.0%)	0.137
Un-integrity	37 (77.1%)	29 (63.0%)	
Time of follow-up (months)	3.7 ± 1.6	3.5 ± 0.9	0.516
Discontinuation of OMT (yes)	5 (10.4%)	3 (6.5%)	0.759

*NBP* Dl-3-butylphthalide, *TIA* transient ischemic attack, *MRA* magnetic resonance angiography, *CTA* computed tomography angiography, *DSA* digital subtraction angiography, *ICA* internal carotid artery, *MCA* middle cerebral artery, *CoW* circle of Willis, *OMT* optimized medical therapy

### rCBF outcomes for ROI analysis

**Fig. 2:**
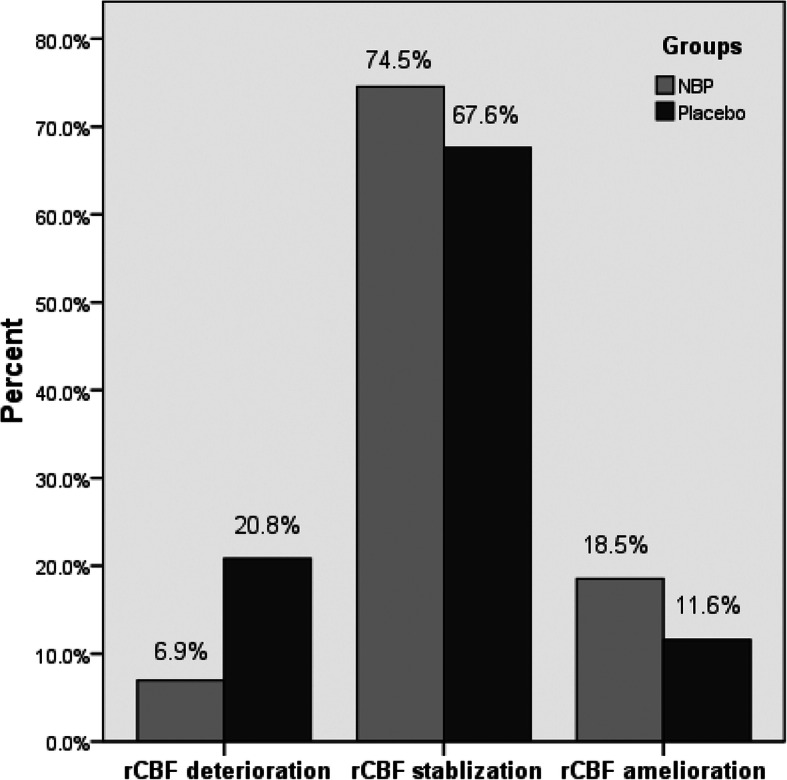
Comparison of rCBF outcomes in every ROI between NBP and placebo groups. There were higher percentages of ROIs with rCBF amelioration and stablization in NBP group, but higher percentages of ROIs with rCBF deterioration in placebo group (*p* = 0.000). CBF, cerebral blood flow; rCBF, regional CBF; ROI, regions of interest; NBP, Dl-3-butylphthalide

Six patients had bilateral carotid artery stenosis and cerebral hypoperfusion among 48 NBP patients, whereas 8 patients did among 46 placebo patients. Therefore, both NBP and placebo groups had a total of 54 stenotic carotid arteries with cerebral hypoperfusion in ipsilateral MCA respectively. Since there were 4 ROIs in the MCA, both groups had 54 × 4 = 216 ROIs for rCBF change analysis respectively. In NBP group, the rCBF in the ROIs significantly increased after treatment (83.5 ± 11.4% vs. 85.8 ± 12.5%, *p* = 0.000). However, no significant change was found in placebo group (86.9 ± 11.6% vs. 87.8 ± 11.7%, *p* = 0.331). In NBP group, the numbers of ROIs with rCBF deterioration, stabilization and amelioration were 15(6.9%), 161(74.5%) and 40(18.5%) respectively, whereas in placebo group, the related numbers were 45(20.8%), 126(67.6%) and 25(11.6%) respectively. Compared with placebo group, significantly higher percentages of ROIs with rCBF amelioration and stabilization were found in NBP group ((93.1% vs. 79.2%, *p* = 0.000, Fig. 2).

### Whole CBF outcomes in ipsilateral MCA for patient analysis

**Fig. 3:**
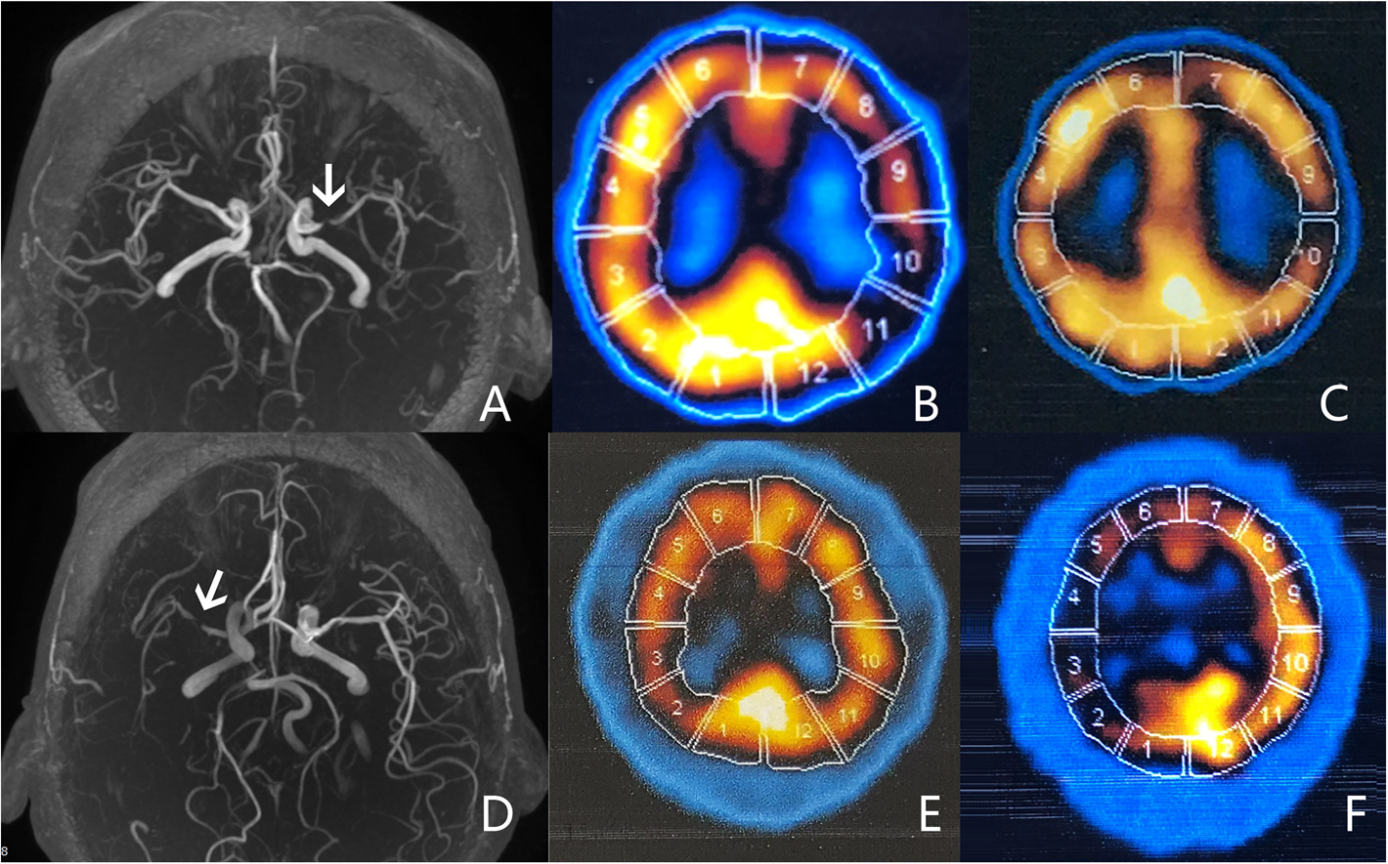
The rCBF change in the ipsilateral MCA after therapy in two patients with carotid artery stenosis shown by MRA. The upper figures showed a patient in NBP group. **a** MRA image showed that the patient had a severe stenosis in the left MCA (white arrow). **b** SPECT image showed that the rCBF in the ipsilateral MCA territory (8–11 ROIs) was impaired before treatment (decreased tracer uptake compared to mirror). **c** SPECT image showed that the decreased rCBF became improved after treatment (increased tracer uptake compared to B). The lower figures showed a patient in placebo group. **d** MRA image showed that the patient had a severe stenosis in the right MCA (white arrow). **e** SPECT image showed that the rCBF in the ipsilateral MCA territory (2–5 ROIs) was impaired before treatment (decreased tracer uptake compared to mirror). **f** SPECT image showed that the decreased rCBF became further deteriorated after treatment (decreased tracer uptake compared to **e**). CBF, cerebral blood flow; rCBF, regional CBF; MCA, middle cerebral artery; MRA, magnetic resonance angiography; NBP, Dl-3-butylphthalide; SPECT, single photon emission computed tomography; ROI, regions of interest

**Fig. 4:**
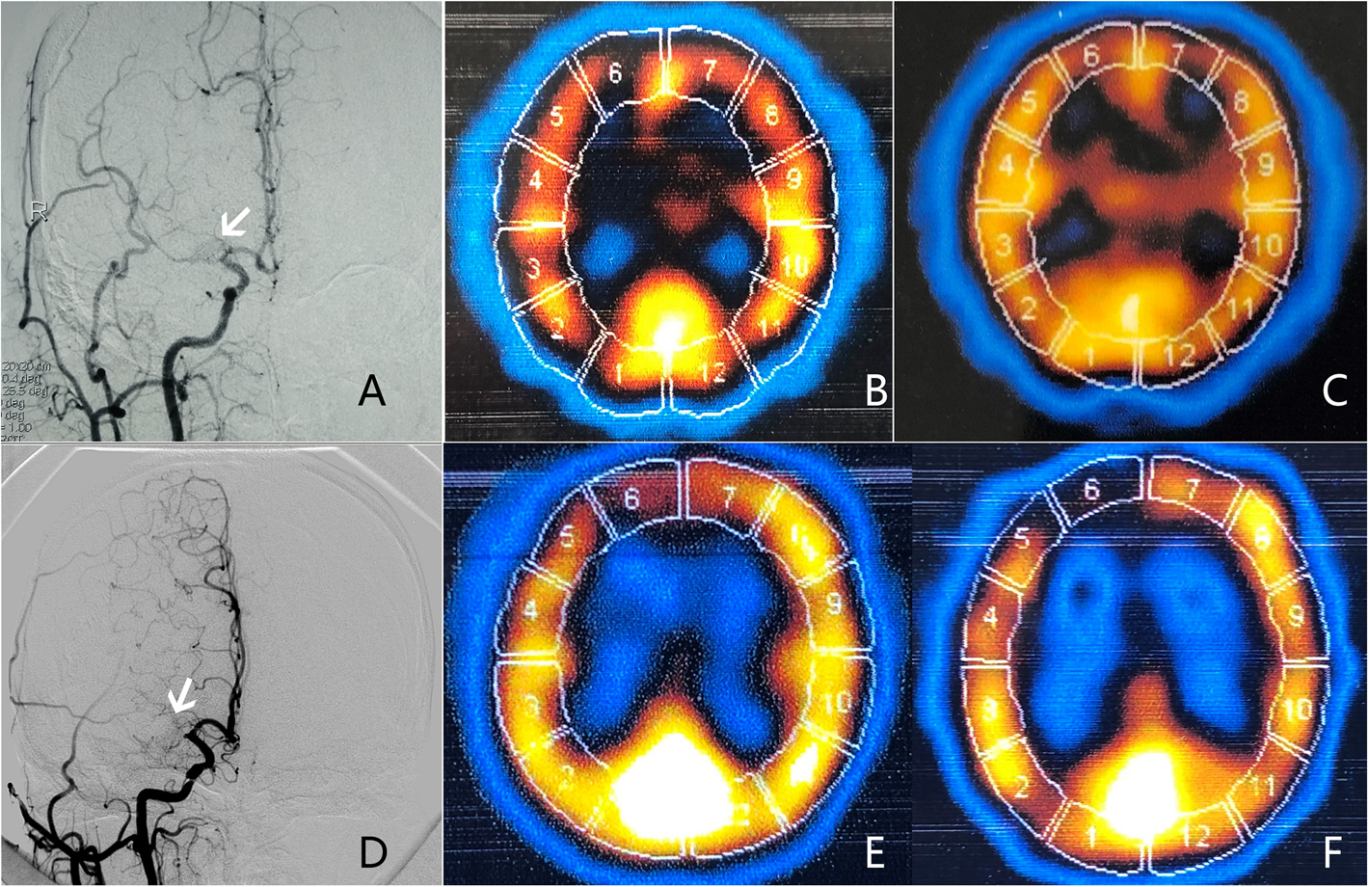
The rCBF change in the ipsilateral MCA after therapy in two patients with carotid artery occlusion shown by DSA. The upper figures showed a patient in NBP group. **a** DSA image showed that the patient had a near-occlusion in the right MCA (white arrow). **b** SPECT image showed that the rCBF in the ipsilateral MCA territory (2–5 ROIs) was impaired before treatment (decreased tracer uptake compared to mirror). **c** SPECT image showed that the decreased rCBF became improved after treatment (increased tracer uptake compared to B). The lower figures showed a patient in placebo group. **d** DSA image showed that the patient had a complete occlusion in the right MCA (white arrow). **e** SPECT image showed that the rCBF in the ipsilateral MCA territory (2–5 ROIs) was impaired before treatment (decreased tracer uptake compared to mirror). **f** SPECT image showed that the decreased rCBF became further deteriorated after treatment (decreased tracer uptake compared to **e**). CBF, cerebral blood flow; rCBF, regional CBF; MCA, middle cerebral artery; DSA, digital subtraction angiography; NBP, Dl-3-butylphthalide; SPECT, single photon emission computed tomography; ROI, regions of interest

No matter how the whole CBF outcomes (deterioration, amelioration and stabilization) in the ipsilateral MCA territory was defined, the higher percentages of patients with whole CBF amelioration and stabilization were always presented in the NBP group, but not in the placebo group (Table 2). When the whole CBF amelioration/deterioration was defined as the numbers of ROIs with rCBF amelioration/deterioration in the patients ≥1, no significant difference in whole CBF outcomes was found between the two groups. However, when the whole CBF amelioration/deterioration was defined as the numbers of ROIs with rCBF amelioration/deterioration in the patients ≥2, ≥3 or ≥ 4, significant differences presented in the whole CBF outcomes between the two groups (Table 2). Notably, due to insufficient data the regression analysis was unable to be performed when the numbers of ROIs were defined as ≥3 and ≥ 4. Finally, the number of ROIs ≥2 was defined as the whole CBF amelioration/deterioration, and be used to analyze the regression. The ordinal regression analysis showed that after adjusted for demographics, atherosclerotic risk factors, bilateral stenosis, stenotic degree, integrity of CoW and discontinuation of OMT, NBP was still an independent factor to improve and stabilize impaired CBF caused by carotid artery stenosis (Table 3). Figures 3 and 4 showed the classical cases whose rCBF in the ipsilateral MCA increased in NBP group and decreased in placebo group. Moreover, when the sample was stratified by carotid stenotic degree (Table 4), the similar trend was also observed in the patients with severe stenosis (*P* = 0.009) and with occlusion (*P* = 0.037). Compared with placebo group, NBP group had higher percentage of whole CBF amelioration in the patients with occlusion, whereas higher percentage of whole CBF stabilization in the patients with severe stenosis.

**Table 2:** Comparisons of whole CBF outcomes in ipsilateral MCA territory of carotid artery system stenosis between NBP and placebo groups

Number of ROIs with rCBF amelioration/deterioration	Group	Deterioration n (%)	Stablization n (%)	Amelioration n (%)	χ2	*P* values
≥1	NBP	10 (20.8%)	23 (47.9%)	15 (31.2%)	2.049	0.359
	Control	15 (32.6%)	21 (45.7%)	10 (21.7%)		
≥2	NBP	1 (2.1%)	37 (77.1%)	10 (20.8%)	11.362	0.003
	Control	12 (26.1%)	27 (58.7%)	7 (15.2%)		
≥3	NBP	1 (2.1%)	40 (83.3%)	7 (14.6%)	8.799	0.012
	Control	10 (21.7%)	31 (67.4%)	5 (10.9%)		
≥4	NBP	0 (0.0%)	43 (89.6%)	5 (10.4%)	6.441	0.015
	Control	5 (10.9%)	39 (84.8%)	2 (4.3%)		

Definitions of whole CBF outcomes were according to the number of ROIs with rCBF amelioration/deterioration. *CBF* cerebral blood flow, *MCA* middle cerebral artery, *NBP* Dl-3-butylphthalide, *ROI* regions of interest, *rCBF* regional CBF

**Table 3:** Odds ratios of NBP vs. placebo for whole CBF outcomes in ipsilateral MCA territory of carotid artery system stenosis

Models	Adjusted factors	Wald-χ^2^	*P* values	OR(95%CI)
1	None	6.080	0.014	3.23 (1.02–8.19)
2	Demographics and atherosclerotic risk factors	5.687	0.017	3.38 (1.24–9.19)
3	Demographics, atherosclerotic risk factors, cerebral stenotic characteristics and integrity of CoW	5.247	0.022	3.31 (1.19–9.20)
4	Demographics, atherosclerotic risk factors, cerebral stenotic characteristics and integrity of CoW, OMT	5.110	0.024	3.26 (1.17–9.06)

*NBP* Dl-3-butylphthalide, *CBF* cerebral blood flow, *MCA* middle cerebral artery, *CoW* circle of Willis. Demographics included age and sex; Atherosclerotic risk factors included hypertention, diabetes mellitus, hyperlipemia, coronary disease and smoking; Cerebral stenotic characteristics included bilateral stenosis and stenotic degree. OMT = optimized medical therapy

**Table 4:** Comparisons of whole CBF outcomes in ipsilateral MCA territory of carotid artery system stenosis between NBP and placebo groups stratified by stenotic degree

whole CBF outcomes	Severe stenosis (*n* = 75)			Occlusion (*n* = 19)		
	NBP	placebo	*P* values	NBP	placebo	*P* values
Deterioration n (%)	1 (2.9%)	11 (27.5%)	0.009	0 (0%)	1 (16.7%)	0.037
Stabilization n (%)	29 (82.9%)	22 (55.0%)		8 (61.5%)	5 (83.3%)	
Amelioration n (%)	5 (14.3%)	7 (17.5%)		5 (38.5%)	0 (0%)	

*CBF* cerebral blood flow, *MCA* middle cerebral artery, *NBP* Dl-3-butylphthalide; The whole CBF amelioration/deterioration was defined as the numbers of ROIs with rCBF amelioration/deterioration in the patients ≥2

## Discussion

Our randomized, double-blind, placebo-controlled trial showed that four weeks of orally NBP therapy improved the rCBF in the ipsilateral MCA of carotid artery stenosis. There were higher percentages of ROIs with rCBF amelioration and stablization after treatment in NBP group than in placebo group. Compared with placebo group, NBP group had more than two times of patients who had the whole CBF amelioration in the ipsilateral MCA.

BNP was licensed as a novel drug for clinical trials in 2002. Based on the results from the multicenter phase 2 and 3 randomized controlled clinical trials, which consistently reported that 21-day or 90-day orally or intravenously NBP therapy could improve neurological deficits and activity of daily life (ADL) scoring after acute ischemic stroke, with a good safety and tolerability, NBP was approved as a therapeutic drug for ischemic stroke by the State Food and Drug Administration of China (SFDA) in 2005 [9, 10]. NBP was recommended to treat the acute ischemic stroke in 2018 Chinese stroke guidelines [15]. A multicentre, randomized, double-blind, placebo-controlled trial recently showed that a 6-month NBP treatment was also effective for improving cognitive and global functioning in the patients with subcortical vascular cognitive impairment no dementia (VCIND) [18]. Another retrospective case-control study showed that postoperative administration of 7-day NBP injection was able to alleviate perioperative neurological deficits after revascularization surgery for the patients with moyamoya disease [19]. NBP might play the positive effects on these diseases through the mechanism of improving the cerebral hypoperfusion [9, 10], since it had been demonstrated that the ischemic stroke, vascular cognitive impairment and perioperative neurological deficits of moyamoya disease after revascularization surgery were partially attributed to acute, chronic or fluctuating cerebral hypoperfusion [1, 5, 19]. An animal study had showed that NBP increased cerebral microvessels and perfusion, and decreased the volume of the cerebral infarct in the rats of cold-induced ischemic stroke [14]. NBP also accelerated CBF in CCH rats after permanent bilateral common carotid artery occlusion, and prevented CCH-induced white matter damage and cognitive impairment [11, 13]. In addition, NBP was found to improve rCBF in the caudate nucleus for the rats subjected to subarachnoid hemorrhage [20]. By contrast, high-quality clinical trial was lack to demonstrate that NBP increased CBF and improved cerebral hypoperfusion.

Our study firstly demonstrated that NBP could improve the cerebral hypoperfusion in the patients with carotid artery atherosclerotic stenosis. However, some important points should be noted to explain and apply this result. First, all the patients in this study had decreased CBF before the treatment because of severe carotid artery stenosis/occlusion and insufficient collateral circulation, which suggested that the hemodynamic impairment existed distal to cerebral artery stenosis. In addition, our study excluded the patients who had acute ischemic cerebral vascular diseases before the trial, which suggested that the patients included in this study had chronic cerebral hypoperfusion. Therefore, this trial provided the new therapy method for the patients with CCH resulted from the carotid artery stenosis [1, 5, 19]. Second, NBP was used with the combination of OMT against atherosclerotic ischemic stroke in this study, because these patients had the large intracranial or extracranial stenosis. Therefore, the effect of NBP on improving CCH in the patients with the cerebral artery stenosis must base on the anti-atherosclerosis OMT, but not NBP alone [21]. Third, NBP was orally taken for 4 weeks in our study, but the second CBF examination was carried out after 12 weeks from the trial beginning in consideration of the radioactivity of the SPECT test. Therefore, this positive result suggested that NBP had a persistent effect on improving the cerebral hypoperfusion in the patients with the cerebral artery stenosis. At last, although NBP had a positive effect on the cerebral perfusion impairment whether it was caused by severe stenosis or occlusion, NBP primarily improved the cerebral perfusion for the patients with occlusion and preferably prevented the cerebral perfusion worsening for the patients with severe stenosis. Therefore, NBP might be more effective in increasing cerebral perfusion for the patients with occlusion by the improvement of collateral flow, assuming the occlusive vessels would continue to be occluded.

The collateral circulation of the brain may be defined as the artery-to-artery anastomotic pathways that are capable, when needed, of supplying nutrient perfusion to a brain region whose primary source of blood flow has been reduced or compromised by disease [22]. In humans, it is generally recognized that there are three pathways of cerebral collaterals. The brain’s primary collateral pathway consists of the CoW-the anastomotic array of arteries at the base of the brain that connect the anterior and posterior portions of the cerebral circulation. The brain’s secondary collateral pathways can be accessed through the ophthalmic artery and leptomeningeal vessels when collateral flow through the CoW is inadequate. The additional collateral pathway also includes the neovascularization [22, 23]. One clinic study showed that NBP injection increased much more brain’s primary and secondary collaterals than the control injection in the patients of acute ischemic stroke [24]. The animal studies demonstrated that NBP promoted the new angiogenesis in the rats of acute ischemic stroke or CCH [11, 12, 25]. Therefore, these results supported the opinion that NBP could improve the cerebral hypoperfusion in the patients with cerebral artery stenosis by increasing the brain’s collateral circulation. The mechanisms that NBP increases the brain’s collateral circulation may be as follows. First, nitric oxide (NO) released from vascular endothelium was thought to participate in arteriogenesis and vasodilation and increase the cerebral collateral. NBP significantly increased the activity of NOS and production of extracellular NO in bovine aortic endothelial cells and bovine cerebral endothelial cells, which might improve the cerebral microcirculation and restore the supply of oxygen and nutrients to ischemic hemisphere [9, 22]. Second, NBP also promote the angiogenesis or revascularization by up-regulating the expressions of various angiogenic factors, such as vascular endothelial growth factor (VEGF), basic fibroblast growth factor (bFGF) and transforming growth factor-β1 (TGF-β1) [12, 25–27], or by regulating some signaling axes or pathways [28, 29].

Since NBP smelled plant odor, the placebo medicine was designed as a low and ineffective dose (10 mg NBP per pill) rather than a blank one, in order to make sure of an effective double-blind in this trial. However, there were also some limitations in this study. First, this study was a single-center trial, and had a relatively small sample size and a little high lost rate of follow-up. Therefore, a multiple-center and large sample size of trial needs to verify the results in this study. Second, some patients in this study completed the CBF examination more than 12 weeks after treatment. Even so, the results may not be affected by this follow-up prolongation because there was no significant difference in the follow-up time between the NBP group and placebo group. Third, collateral vessels were not directly evaluated in this study. Therefore, this study could not confirm that NBP definitely improved the cerebral hypoperfusion through a mechanism of collateral circulation. However, it is a very interesting point to study in the future.

## Conclusions

Our trial showed that on the basis of OMT, NBP improved the cerebral hypoperfusion in the patients with carotid artery atherosclerotic stenosis. This study provides a new therapy strategy to prevent ischemic stroke and vascular cognitive impairment due to cerebral large-vessel stenosis and hypoperfusion.

## Data Availability

The datasets used and/or analyzed during the current study are available from Clinical Trial Management Public Platform and ResMan research manager and the registration is required to access this data. (http://www.medresman.org.cn/login.aspx).

## Abbreviations

CEA: Carotid endarterectomy
CAS: Carotid artery stenting
NBP: Dl-3-butylphthalide
CBF: Cerebral blood flow
MCA: Middle cerebral artery
PLA: People’s Liberation Army
ICA: Internal carotid artery
TIA: Transient ischemic attacks
OMT: Optimized medical therapy
CoW: Circle of Willis
MRA: Magnetic resonance angiography
CTA: Computed tomography angiography
DSA: Digital subtraction angiography
NASCET: North American Symptomatic Carotid Endarterectomy Trial Collaborators
WASID: Warfarin-Aspirin Symptomatic Intracranial Disease
SPECT: Single photon emission computed tomography
ROI: Region of interest
ECD: Ethylcysteinate dimer
OML: Orbitomeatal line
ADL: Activity of daily life
SFDA: State Food and Drug Administration
VCIND: Subcortical vascular cognitive impairment no dementia
CCH: Chronic cerebral hypoperfusion
NO: Nitric oxide
VEGF: Vascular endothelial growth factor
bFGF: Basic fibroblast growth factor
TGF-β1: Transforming growth factor-β1

## References

[1] Banerjee C, Turan TN (2017). Large artery atherosclerosis: Extracranial and intracranial. Semin Neurol.

[2] Shakur SF, Hrbac T, Alaraj A, Du X, Aletich VA, Charbel FT (2014). Effects of extracranial carotid stenosis on intracranial blood flow. Stroke..

[3] Mozaffarian D, Benjamin EJ, Go AS, Arnett DK, Blaha MJ, Cushman M, Writing Group Members (2016). Heart disease and stroke statistics—2016 update: a report from the American Heart Association. Circulation..

[4] Xu J, Liu L, Wang Y, Zhao X, Wang C, Wang A (2012). TOAST subtypes:its influence upon doctors' decisions of antihypertensive prescription at discharge for ischemic stroke patients. Patient Prefer Adherence.

[5] Naylor AR, Ricco JB, de Borst GJ, Debus S, de Haro J, Halliday A (2018). Editor's choice - Management of Atherosclerotic Carotid and Vertebral Artery Disease: 2017 clinical practice guidelines of the European Society for Vascular Surgery (ESVS). Eur J Vasc Endovasc Surg.

[6] Chen DW, Zheng J, Shi J, Yin YW, Song C, Yang F (2018). Assessment of the Cerebral Hemodynamic Benefits of Carotid Artery Stenting for Patients with Preoperative Hemodynamic Impairment Using Cerebral Single Photon Emission Computed Tomography (SPECT) and Carbon Dioxide Inhalation. Med Sci Monit.

[7] Nishijima Y, Akamatsu Y, Weinstein PR, Liu J (2015). Collaterals: implications in cerebral ischemic diseases and therapeutic interventions. Brain Res.

[8] Powers WJ, Rabinstein AA, Ackerson T, Adeoye OM, Bambakidis NC, Becker K (2019). Guidelines for the Early Management of Patients With Acute Ischemic Stroke: 2019 Update to the 2018 Guidelines for the Early Management of Acute Ischemic Stroke: A Guideline for Healthcare Professionals From the American Heart Association/American Stroke Association. Stroke..

[9] Wang S, Ma F, Huang L, Zhang Y, Peng Y, Xing C (2018). Dl-3-n-Butylphthalide (NBP): a promising therapeutic agent for ischemic stroke. CNS Neurol Disord Drug Targets.

[10] Xu ZQ, Zhou Y, Shao BZ, Zhang JJ, Liu C (2019). A systematic review of Neuroprotective efficacy and safety of DL-3-N-Butylphthalide in ischemic stroke. Am J Chin Med.

[11] Xiong Z, Lu W, Zhu L, Zeng L, Shi C, Jing Z (2017). Dl-3-n-Butylphthalide treatment enhances hemodynamics and ameliorates memory deficits in rats with chronic cerebral Hypoperfusion. Front Aging Neurosci.

[12] Zhang L, Lü L, Chan WM, Huang Y, Wai MS, Yew DT (2012). Effects of DL-3-n-butylphthalide on vascular dementia and angiogenesis. Neurochem Res.

[13] Han QY, Zhang H, Zhang X, He DS, Wang SW, Cao X (2019). dl-3-n-butylphthalide preserves white matter integrity and alleviates cognitive impairment in mice with chronic cerebral hypoperfusion. CNS Neurosci Ther.

[14] Liu CL, Liao SJ, Zeng JS, Lin JW, Li CX, Xie LC (2007). dl-3n-butylphthalide prevents stroke via improvement of cerebral microvessels in RHRSP. J Neurol Sci.

[15] Chinese Society of Neurology, Chinese Stroke Society (2018). Chinese guidelines for diagnosis and treatment of acute ischemic stroke 2018. Chin J Neurol.

[16] Kim HJ, Kim TW, Ryu SY, Yang PS, Kwon MJ, Kim JC (2011). Acetazolamide-challenged perfusion magnetic resonance imaging for assessment of cerebrovascular reserve capacity in patients with symptomatic middle cerebral artery stenosis: comparison with technetium-^99^m-hexamethylpropyleneamine oxime single-photon emission computed tomography. Clin Imaging.

[17] Pei-li C, Jin S, Wei-qing Z, Jin Z, Ying-qian Z, Lu Q (2017). Change of cerebral perfusion parameters in patients with middle cerebral artery stenosis during a short-term follow-up time. Chin J Geriatr Heart BrainVessel Dis.

[18] Jia J, Wei C, Liang J, Zhou A, Zuo X, Song H (2016). The effects of DL-3-n-butylphthalide in patients with vascular cognitive impairment without dementia caused by subcortical ischemic small vessel disease: A multicentre, randomized, double-blind, placebo-controlled trial. Alzheimers Dement.

[19] Li Z, Lu J, Ma L, Wu C, Xu Z, Chen X (2019). dl-3-n-butylphthalide for alleviation of neurological deficit after combined extracranial-intracranial revascularization for moyamoya disease: a propensity score-matched analysis. J Neurosurg.

[20] Chong ZZ, Feng YP (1999). dl-3-n-butylphthalide improves regional cerebral blood flow after experimental subarachnoid hemorrhage in rats. Zhongguo Yao Li Xue Bao.

[21] Spence JD, Naylor AR (2016). Endarterectomy, stenting, or neither for asymptomatic carotid-artery stenosis. N Engl J Med.

[22] Ginsberg MD (2018). The cerebral collateral circulation: Relevance to pathophysiology and treatment of stroke. Neuropharmacology.

[23] Liu J, Wang Y, Akamatsu Y, Lee CC, Stetler RA, Lawton MT (2014). Vascular remodeling after ischemic stroke: mechanisms and therapeutic potentials. Prog Neurobiol.

[24] Wu Y-F, Xiao-Hong L, Wei-Cheng G, Jie Z, Hao-Ran W, Fei-Fei H (2017). Effect. Observation of Butylphthalide injection in treating acute anterior circulation infarction. Chin J Stroke.

[25] Liao SJ, Lin JW, Pei Z, Liu CL, Zeng JS, Huang RX (2009). Enhanced angiogenesis with dl-3n-butylphthalide treatment after focal cerebral ischemia in RHRSP. Brain Res.

[26] Zhou PT, Wang LP, Qu MJ, Shen H, Zheng HR, Deng LD (2019). Dl-3-N-butylphthalide promotes angiogenesis and upregulates sonic hedgehog expression after cerebral ischemia in rats. CNS Neurosci Ther..

[27] Jiang Y, Sun L, Xuan X, Wang J (2016). Impacts of N-Butylphthalide on expression of growth factors in rats with focal cerebral ischemia. Bosn J Basic Med Sci.

[28] Li W, Wei D, Xie X, Liang J, Song K, Huang L (2019). Dl-3-n-Butylphthalide regulates the Ang-1/Ang-2/Tie-2 signaling axis to promote neovascularization in chronic cerebral hypoperfusion. Biomed Pharmacother.

[29] Lu XL, Luo D, Yao XL, Wang GL, Liu ZY, Li ZX (2012). dl-3n-Butylphthalide promotes angiogenesis via the extracellular signal-regulated kinase 1/2 and phosphatidylinositol 3-kinase/Akt-endothelial nitric oxide synthase signaling pathways. J Cardiovasc Pharmacol.

